# 

**Published:** 1896-10-17

**Authors:** 


**Notices of Births, Deaths, and Marriages** are inserted in this column at a charge of 2s. 6d. for an announcement not exceeding four lines.

### NOTICE TO CORRESPONDENTS.

All MS., Letters, Books for Review, and other matters intended for the Editor should be addressed The Editor, The Hospital Building, 28 & 29, Southampton Street, Strand, W.C.
The Editor cannot undertake to return rejected MS., even when accompanied by stamped directed envelope.

**Notice to Advertisers and Subscribers.**

All Advertisements, Orders for Copies of The Hospital, and Business Communications should be addressed to **THE MANAGER, (Not to The Editor),** The Hospital, 28 & 29, Southampton Street, Strand, London, W.C.
The Cost of Subscription, post free, is as follows:—Per week, 2½d.; per quarter, 2s. 8d.; per half-year, 5s. 3d.; per annum, 10s. 6d. Foreign:—Per annum, 15s. 2d.; payable, in all cases, in advance.

**IMPORTANT NOTICE.**

The EDITORIAL and PUBLISHING OFFICES of "THE HOSPITAL" are now TRANSFERRED to their new premises, 28 & 29, SOUTHAMPTON STREET, STRAND, LONDON, W.C.

**NOTICE.—Vol. XX. of "The Hospital" (April to September, 1896), in handsome cloth binding, is now on sale at the Office of this Journal, price 7s. 6d. Cases for binding the Numbers contained in this Volume 1s. 6d. Index to Vol. XX., 2½d., post free. The Monthly Part for October is now ready, Price 6d., by post 8d.**

### SCRAPS AND GLEANINGS.

Miss Georgiana E. Ormerod has bequeathed £200 to the Dental Hospital of London.

Upwards of £800 was realised by the recent concert at Cardiff. The amount is to be divided between the Cardiff Infirmary and the poor of Craig-y-nos.

The second annual charity demonstration at Arbroath, resulted in a sum of £82 odd, which is to be divided amongst the local charities, viz., Arbroath Infirmary, Convalescent Home, District Nursing Association, and the Lifeboat Association.

The Foresters' Convalescent Home, at Clent, has been enriched recently by £130 3s. 10d., being the proceeds of the fête held on behalf of this institution. This sum, together with £100 given by Mr. J. H. Cheshire, and about £70 collected from various sources, is to clear off the debt on the Clent Forester's Convalescent Home.

### The Student of Medicine.

### APPOINTMENTS.

H. B. Angus, M.B., M.R.C.S., appointed Honorary Assistant Surgeon to the Royal Infirmary, Newcastle-on-Tyne. Dr. Brownlees, appointed Medical Officer to the Glenwherry Dispensary. A. H. Card, M.R.C.S., L.R.C.P., appointed Surgeon, &c., to the Worksop Dispensary. Wm. H. Coates, M.A., M.B.Durh., L.R.C.P.Lond., M.R.C.S.Eng., appointed Medical Officer of Health to the Patrington Rural District Council and Medical Officer to the Patrington Workhouse. P. W. Diack, M.A., M.B., C.M.Aberd., appointed Senior House-Surgeon to the Blackburn and East Lancashire Infirmary. C. F. Fenton, L.R.C.P.Lond., M.R.C.S.Eng., appointed Medical Officer of Health for the Barking Town Urban District. L. Glover, M.A.Camb., M.B., M.R.C.S., L.R.C.P.Lond., appointed Assistant Medical Officer with Charge of the Out-patients at the Hampstead Hospital. A. Graham, M.B., appointed Medical Officer for the Seventh District of the Newmarket Union. F. Hawthorn, M.D., B.S.Durh., M.R.C.S.Eng., L.R.C.P.Lond., appointed Resident Medical Officer to the Dispensary, Newcastle-on-Tyne. S. Hayes, M.D.Lond., appointed Anæsthetist to the General Hospital, Birmingham. C. A. Leedham-Green, F.R.C.S.Eng., M.D.Heidlg., appointed Anæsthetist to the General Hospital, Birmingham. F. D. S. McMahon, L.R.C.P., L.R.C.S.Edin., appointed Medical Officer for the Parishes of St. Columb, St. Wenn, St. Newgan, and St. Eval of the St. Columb Union. R. H. Mornement, M.R.C.S.Eng., L.R.C.P.Lond., appointed Assistant Medical Officer at the London County Asylum, Cane Hill, Purley, Surrey. Mary C. Murdoch, L.R.C.P. & S.Edin., appointed Assistant Physician to the Victoria Hospital for Children, Hull. A. Nobbs, M.B.Edin., appointed Medical Officer for the No. 1 District of the Battersea, Clapham, and Wandsworth Union. R. Roche, B.A., M.R.C.S., L.R.C.P.Lond., appointed Medical Officer to and Lecturer on Hygiene, Ambulance, and Domestic Medicine at the Colonial College, Hollesley Bay. E. W. St V. Ryan, L.R.C.P., L.R.C.S.Edin., L.F.P.S.Glasg., appointed Medical Officer for the No. 5 District of the Battersea, Clapham, and Wandsworth Union. E. C. Taylor, M.D.Lond., F.R.C.S.Eng., appointed House Surgeon to the Bristol Hospital for Sick Children and Women. W. J. Thompson, M.D.Dub.Univ., appointed Visiting Physician to Jervis Street Hospital, Dublin. H. R. Wadd, M.R.C.S.Eng., L.R.C.P.Lond, late Junior House Surgeon, appointed Senior House Surgeon to the Great Northern Central Hospital, N.

### PASS LIST.

**The Royal University of Ireland.**

Third Examination in Medicine.

The following candidates have satisfied the examiners:—

Upper Pass.—R. T. Booth, Queen's College, Cork; P. Canning, M.A., Catholic University School of Medicine; D. McCay, Queen's Colleges, Cork and Belfast; A. B. McMaster, Queen's College, Belfast; E. C. T. Smith, Mason College, Birmingham. The above candidates may present themselves for the further examination for honours:—

Pass.—Dora E. Allman, Queen's College, Cork; D. Brown, Queen's College, Belfast; W. Cahill, Queen's College, Cork; H. L. Craig, Queen's College, Belfast; J. Dorgan, Queen's College, Cork; F. Fulton, Queen's College, Belfast; W. Hartnett, Queen's College, Cork; G. Jefferson, Queen's College, Belfast; S. McCann, Catholic University School of Medicine; D. Mahony, Catholic University School of Medicine; J. Martin, Queen's College, Belfast; D. Murphy, Queen's College, Cork; J. Murray, Catholic University School of Medicine; W. Paisley, Queen's College, Galway; R. L. Patterson, Queen's College, Belfast; P. M. Quinn, Catholic University School of Medicine; W. J. Shannon, B.A., Queen's College, Belfast; R. A. Shaw, Queen's College, Belfast; Frances O. C. Sinclair, B.A., School of Medicine for Women, Edinburgh; J. B. Slattery, Queen's College, Cork; Lucy E. Smith, Queen's College, Cork; W. J. Sweeny, School of Physic, Trinity College, Dublin, and Royal College of Surgeons; W. White, Queen's College, Cork; J. E. Whyte, Queen's College, Belfast.

